# Sampling beetle communities: Trap design interacts with weather and species traits to bias capture rates

**DOI:** 10.1002/ece3.7029

**Published:** 2020-11-18

**Authors:** Ryan C. Burner, Tone Birkemoe, Siri Lie Olsen, Anne Sverdrup‐Thygeson

**Affiliations:** ^1^ Faculty of Environmental Sciences and Natural Resource Management Norwegian University of Life Sciences Ås Norway; ^2^ Norwegian Institute for Nature Research Oslo Norway

**Keywords:** Bayesian methods, interaction, linear models, modeling (Community Ecology), monitoring (Community Ecology), monitoring (Population Ecology), sampling

## Abstract

Globally, many insect populations are declining, prompting calls for action. Yet these findings have also prompted discussion about sampling methods and interpretation of long‐term datasets. As insect monitoring and research efforts increase, it is critical to quantify the effectiveness of sampling methods. This is especially true if sampling biases of different methods covary with climate, which is also changing over time. We assess the effectiveness of two types of flight intercept traps commonly used for beetles, a diverse insect group responsible for numerous ecosystem services, under different climatic conditions in Norwegian boreal forest. One of these trap designs includes a device to prevent rainwater from entering the collection vial, diluting preservatives and flushing out beetles. This design is compared to a standard trap. We ask how beetle capture rates vary between these traps, and how these differences vary based on precipitation levels and beetle body size, an important species trait. Bayesian mixed models reveal that the standard and modified traps differ in their beetle capture rates, but that the magnitude and direction of these differences change with precipitation levels and beetle body size. At low rainfall levels, standard traps catch more beetles, but as precipitation increases the catch rates of modified traps overtake those of standard traps. This effect is most pronounced for large‐bodied beetles. Sampling methods are known to differ in their effectiveness. Here, we present evidence for a less well‐known but likely common phenomenon—an interaction between climate and sampling, such that relative effectiveness of trap types for beetle sampling differs depending on precipitation levels and species traits. This highlights a challenge for long‐term monitoring programs, where both climate and insect populations are changing. Sampling methods should be sought that eliminate climate interactions, any biases should be quantified, and all insect datasets should include detailed methodological metadata.

## Graphical abstract

Many insect populations appear to be in decline, and robust sampling methods are needed to describe population trends. Yet researchers seldom consider how insect capture rates may covary with climate, biasing long‐term data sets. We demonstrate a precipitation‐capture rate interaction in several commonly used trap designs.

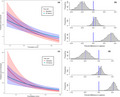

## 1 INTRODUCTION

Globally, many insect populations are in decline (Cardoso et al., 2020; Sánchez‐Bayo & Wyckhuys, 2019; Wagner, 2020), prompting increased public concern as well as calls for action from the scientific community (Lamarre et al., 2020; Samways, 2019). Yet these findings have also prompted much discussion about sampling methods and their relevance for the interpretation of results (Didham et al., 2020; Montgomery et al., 2020; Thomas et al., 2019). Many countries are increasing insect monitoring and research to further understand these declines (Delbrück & Nürnberg, 2019; Norwegian Ministries, 2018). To ensure that these increases in monitoring, as well as corresponding analyses of historic datasets, are robust requires that we understand and quantify differences in effectiveness between sampling methods under different environmental conditions.

Insects are a species‐rich group with a wide diversity of behaviors and habitats. This means that a variety of sampling strategies are required, and most monitoring efforts will focus on certain subsets of taxa (Didham et al., 2020; Lebuhn et al., 2013). In forest ecosystems, beetles are a large and functionally important group that contributes to a range of ecosystem services, like decomposition, pollination, and pest control, in addition to being an important food source for other animals. They also appear to be in decline in the regions where long‐term data have been investigated (Homburg et al., 2019; Seibold et al., 2019).

Forest beetles are most effectively sampled using flight intercept (window) traps (Allison & Redak, 2017; Bouget et al., 2008; Siitonen, 1994). These traps consist of a central, most often transparent, panel (or two crossed panels) with which flying insects collide, a collection bottle below into which these insects then fall, and a roof to keep out rain and debris. Much of what is known about forest insects was gleaned from capture records and specimens caught using these traps (Davies et al., 2008), but designs are not standardized and traps commonly vary in size, shape, color, materials, and the type of preservative used. This is a concern, because there can be major differences in effectiveness between capture methods; this is true for different trap types (Hyvärinen et al., 2006; Siitonen, 1994), but also for slight modifications of very similar flight intercept (Allison & Redak, 2017; Knuff et al., 2019; Rassati et al., 2019; Ulyshen & Sheehan, 2019), pitfall (Woodcock, 2005), and malaise (Åström et al., 2020; Matthews & Matthews, 2017) traps. Capture rates can vary not only due to these differences intrinsic to trap design, but also from subtle differences in placement (e.g., baiting effects, trap height, ambient temperature, openness, and ease of flight) (Flaherty et al., 2019; Økland, 1996; Seibold et al., 2016; Sheehan et al., 2019). Particularly relevant for long‐term monitoring programs are differences in capture rates due to precipitation and temperature; these climatic factors can vary not only with microhabitat of individual trap sites, but also at larger spatial and temporal scales due to climate change.

Furthermore, differences in trap design and placement can affect capture rates of different species in different ways. Estimating species‐specific effects for diverse groups such as insects is difficult, even when data are available to compare trap types, because there are many rare species. Species traits (Seibold et al., 2015), however, can be helpful in predicting the relative effectiveness of different traps and trap conditions for rare species if capture rates of species are correlated with their traits (Garrard et al., 2013).

Apart from ensuring that sampling methodologies are standardized, the biggest practical challenge for large‐scale flight intercept trapping efforts is to maintain functioning traps in remote forest environments (Bouget et al., 2008). This is important, because the large monitoring efforts that are needed to effectively characterize insect trends are expensive and become much more expensive if traps must be actively maintained by repeated visits throughout a season. Rain is a particular challenge due to its propensity to enter traps, dilute preservatives, and make identification by DNA barcoding impossible, and overflow collection bottles and wash away some of the insects. Drainage holes can be made in the collection bottles, but they frequently get blocked. The longer that traps must be left in place without attention, as is often necessitated by large projects, remote areas, and stretched budgets, the more pronounced the problem. This will become an increasingly important challenge (and potential source of bias) over time, given the predictions of wetter summer weather for some forested regions, including northern Europe (Alexander, 2016; Ljungqvist et al., 2019), and as monitoring efforts are expanded in the humid tropics (Montgomery et al., 2020).

Custom trap modifications, which may mitigate these effects of rain but will also be an additional source of methodological variation among datasets, are likely to become more common. One design, a device designed by the Forest Research Institute (Sękocin Stary, Poland), modifies the Polish IBL‐2 trap (CHEMIPAN, Warsaw, Poland) that has been used to sample beetles across Europe (Hägglund et al., 2020; Joelsson et al., 2017; Marczak et al., 2018; Stenbacka et al., 2010; Szafraniec et al., 2019). This rainwater‐filtering device uses a screen and funnel system just above the collection bottle to divert rainwater away while still allowing insects to enter the bottle. To our knowledge, this is the only such type of device currently in use for insect sampling with flight intercept traps, but the effects of these modifications on capture rates, and the resultant effects on long‐term datasets, have not been assessed. In 2018, we began to systematically test whether these rainwater‐filtering devices had any biasing effects on beetle captures relative to the standard traps.

In this study, we placed mixed clusters of standard window traps and window traps with a rainwater draining device in Norwegian boreal forests to test the hypotheses that: (a) capture rates of beetle species differ substantially between the trap types, (b) these differences covary with beetle species traits (body size and indicator status), and (c) the magnitude of these differences depends on rainfall patterns.

## 2 MATERIALS AND METHODS

### 2.1 Insect traps and study sites

**Figure 1:**
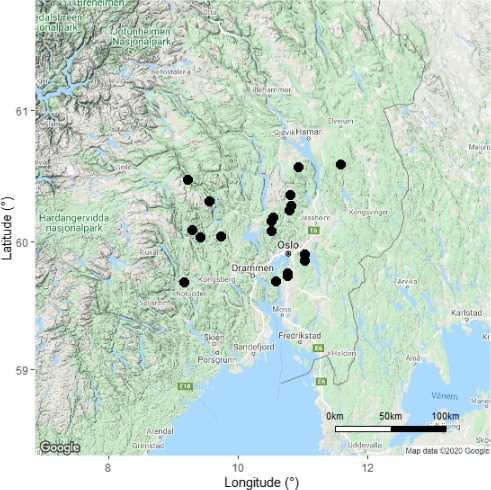
Locations of clusters of flight intercept (window) traps placed to capture forest beetles in 2018 and 2019 in south‐eastern Norway. Five traps were placed at each location, in mature coniferous forest

**Figure 2:**
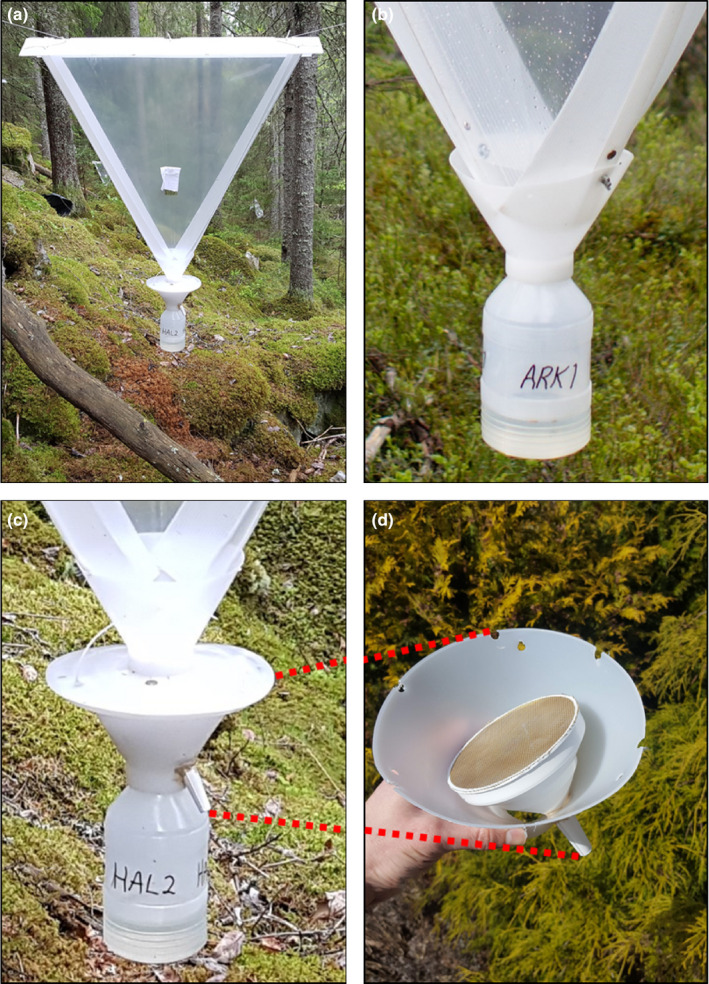
Flight intercept (window) traps placed in Norwegian forests to sample beetle communities (a). These Polish LBL‐2 traps are designed and produced by CHEMIPAN (Warsaw, Poland). Each trap is fitted with a bottle of propylene glycol to preserve insects that are captured. Rainwater often dilutes the glycol and overflows the bottles even when drainage holes are in place. To test a trap modification meant to eliminate this problem, two types of trap were placed at each site: standard traps (b) and traps with a rainwater draining device (c). This device (d), designed by the Department of Forest Protection, Forest Research Institute (Sękocin Stary, Poland), funnels rainwater out of the spout, protected by a screen to retain captured insects. These insects must then crawl around the screen to fall into the collection bottle. Photos by RCB and Roar Økseter

We trapped beetles at 20 sites in coniferous boreal forest in Norway from July to August in 2018 and from June to August in 2019 (Figure 1). The sites were located in near‐natural forest and selected from databases of protected forests and/or forests with high occurrence of red‐listed wood‐living fungi (Naturbase https://www.miljodirektoratet.no/tjenester/naturbase/ and Artskart https://artskart.artsdatabanken.no/). Five triangular single‐plane transparent window traps (90 × 76 cm), including one to two standard traps and three to four traps with water draining devices (Figure 2), were placed at each site. These Polish IBL‐2 traps are produced by CHEMIPAN (Warsaw, Poland) (Pettersson et al., 2007; Stenbacka et al., 2010). The rainwater draining device, designed by the Department of Forest Protection, Forest Research Institute (Sękocin Stary, Poland), contains an internal screen that prevents beetles from escaping through a funnel that diverts rainwater out of the trap. Insects must crawl over the edge of this screen in order to fall into the collection bottle (Figure 2). At each site, traps were placed randomly within a 30‐m radius and hung between trees.

Traps were emptied in the middle of the season (“Period 1”; 2019 only) and again at the end (“Period 2”; 2018 & 2019). We recorded the number of days traps were placed at each site in each trap period. Beetle species captured in each window trap were identified to species level by an expert taxonomist, and the number of individuals of each species was recorded. Beetle records were all uploaded to GBIF (gbif.org). Body size (length) of each species was taken from Seibold et al. (2015) when available (*n* = 420 of 452 species). Relevant species were also classified as natural forest‐indicator species based on a classification system used by Dahlberg (2011).

### 2.2 Precipitation estimates

To estimate rainfall at each of the trap sites during each trap period, we downloaded estimated daily total precipitation values for the study area from Lussana et al. (2019). These values are estimated across Norway at a spatial resolution of 1 × 1 km and are based on a model‐based interpolation. Precipitation values for each site were summed across the days that traps were placed during each trapping period. All analyses were conducted in R (R Core Team, 2019); climate data were extracted using the *raster* (Hijmans, 2019) package. Total precipitation values in our dataset ranged from 38 mm to 228 mm during a mean trap period of 38 days.

### 2.3 Data analysis

To determine the effects of trap type and precipitation on capture rates, we used Bayesian mixed models in the *R2jags* package (Su & Yajima, 2015). We fit models with one of several response variables, including total number of species captured and total number of individuals captured. Counts were overdispersed, necessitating a negative binomial distribution rather than a Poisson. We also fit models with the count of species of several subgroups as response variables to test for differences among these groups: natural forest‐indicator species (*n* = 130 of 452 total species), large‐bodied species (*n* = 104; length > 5.0 mm), and small‐bodied species (*n* = 48; length < 1.5 mm). These body size cutoffs were chosen because they represented natural breaks in the body size distribution histogram. Indicator species were on average 23% shorter than nonindicator species (95% credible interval 0.17–1.51 mm shorter) in our dataset.

Dependent variables included trap type and trapping period (period 1, early summer versus period 2, late summer) as categorical covariates, and trap effort (number of trap days) and total precipitation during the trapping period as continuous covariates. Site was included as a random effect with a random intercept. An interaction term for trap type*precipitation was included as well because we expected the trap types to perform differently at different precipitation levels. The effect of year was not important in preliminary analyses and so year was not included to minimize model complexity. All covariates were scaled (*μ* = 0, *SD* = 1.0) prior to model fitting. A set of six biologically plausible models, with different combinations of the above covariates, was fit for each response variable (Table 1). Models were compared using the deviance information criterion (DIC) (Spiegelhalter et al., 2014).

**Table 1:** Penalized DIC scores to compare Bayesian linear mixed models of the number of beetles caught in a single flight intercept trap. Several response variables, in the right‐hand columns, were used, representing counts of species and individuals of various groups of beetles. All counts were overdispersed and so were modeled using a negative binomial distribution. Two trap types were used—standard traps, and those fitted with a rainwater draining device. Five traps were place at each of 20 sites, and site was included as a random‐intercept effect in each model. Other covariates included the trap period (period 1, early summer versus period 2, late summer), effort (number of trap days), and total precipitation during the capture period. Lowest DIC scores (indicating best model fit, penalized for additional parameters) are indicated in bold font

Model	Response variable				
	Species	Individuals	Large species	Small species	Indicator species
~Trap + Period + Effort + Precipitation + Trap*Precip	**2,143**	**2,984**	**1,371**	1,216	**1,568**
~Trap + Period + Effort + Precipitation	2,157	3,000	1,385	**1,214**	1,574
~Trap + Period + Effort	2,195	3,039	1,390	1,239	1,599
~Period + Effort + Precipitation	2,206	3,042	1,410	1,270	1,615
~Period + Effort	2,194	3,038	1,395	1,239	1,597
~1	2,155	2,998	1,388	1,215	1,572

We assumed that each observed count (i.e., the response variable) was independently distributed according to a negative binomial distribution (for *i* traps), which has two parameters, *p* (probability of success) and *r* (overdispersion parameter):

$${\text{Count}}_{i}∼\text{negbin}\left(p_{i},r\right),\text{where}:p_{i}=r/\left(r+\mu_{i}\right).$$

Here, *μ_i_* denotes the expected mean of the observed count at trap *i* for site *s*. We used a negative binomial regression model with log link to explain the mean parameter,

$$\begin{array}{c} \log\left(\mu_{i}\right)∼\alpha_{s}+\beta_{1}*{\text{trap type}}_{i}+\beta_{2}*\text{trap period}+\beta_{3}\\ {}*\text{effort}+\beta_{4}*{\text{precipitation}\;}_{i}+\beta_{5}*{\text{precipitaion}}_{i}*{\text{trap type}}_{i}, \end{array}$$

In R coding language, the full model formula can be written as:

Count∼traptype+trapperiod+effort+precipitation+traptype:precipitation+(1|Site)

Uninformative priors were set for all parameters; intercepts α_s_ for the random effect of site were drawn from a normal distribution with a prior mean of θ and standard deviation τ, denoted as *N*(θ, τ). The prior parameter θ is given a hyperprior of *N*(0, 100) and τ is given a hyperprior of 1/s^2^, where s has a uniform prior of Unif(0, 100). Each regression coefficient β in the model had a prior of *N*(0, 0.0001). The negative binomial dispersion parameter, *r*, was assumed to have a uniform prior set at Unif(0, 40). All models were fit using 3 chains, 20,000 iterations each, with a burn‐in of 5,000 and thinning of 25. Model convergence was assessed using Gelman and Geweke diagnostics, and by checking effective sample sizes (Boone et al., 2014).

## 3 RESULTS

The full model, with a trap type*precipitation interaction term, performed best for predicting counts of total species, total individuals, number of large‐bodied species, and indicator species, based on penalized DIC scores (Table 1). For number of small‐bodied species, the model with all covariates but without the interaction performed best (ΔDIC = 2). All chains converged adequately in all models (mean Rhat < 1.05).

**Figure 3:**
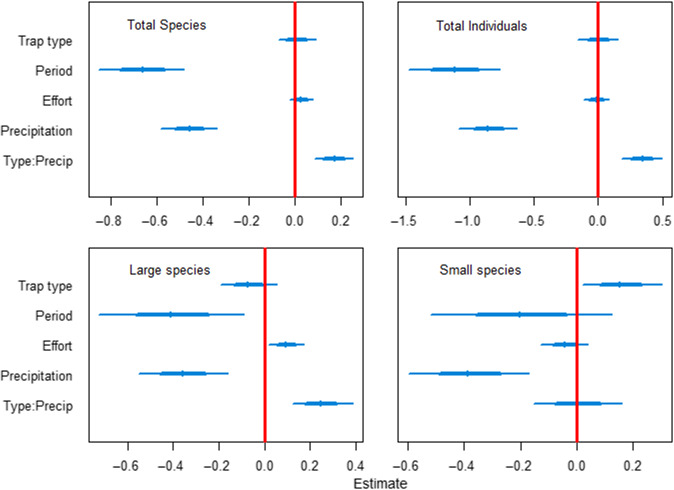
Coefficient estimates for the full model for each of four response variables of beetle captures rates from flight intercept traps placed in Norwegian boreal forests. Thick and thin blue lines show 95% and 68% posterior credible intervals, respectively. Response variables for each model are listed in the corresponding plot: total species, total individuals, number of large‐bodied species (length > 5.0 mm), and number of small‐bodied species (length < 1.5 mm). Five traps were placed in each of 20 sites, and intercepts for the sites were modeled as a random effect. Standard type traps were the intercept trap type, so the trap type parameter is an estimate for the traps that were modified to include a rainwater draining device

In all models, parameter estimates indicate that trap period had the largest effect (Figure 3), with fewer beetles captured later in the summer. Precipitation also had a strong negative effect, and the effect of effort was slightly positive. Trap type did not have an effect, except for small species, but the interaction term for trap type and precipitation had an effect in all cases except for small species, showing that performance of the traps with the rainwater draining devices diverged from that of the standard traps when precipitation was high. Parameter estimates for indicator species are similar to those for all species (Figure S1).

**Figure 4:**
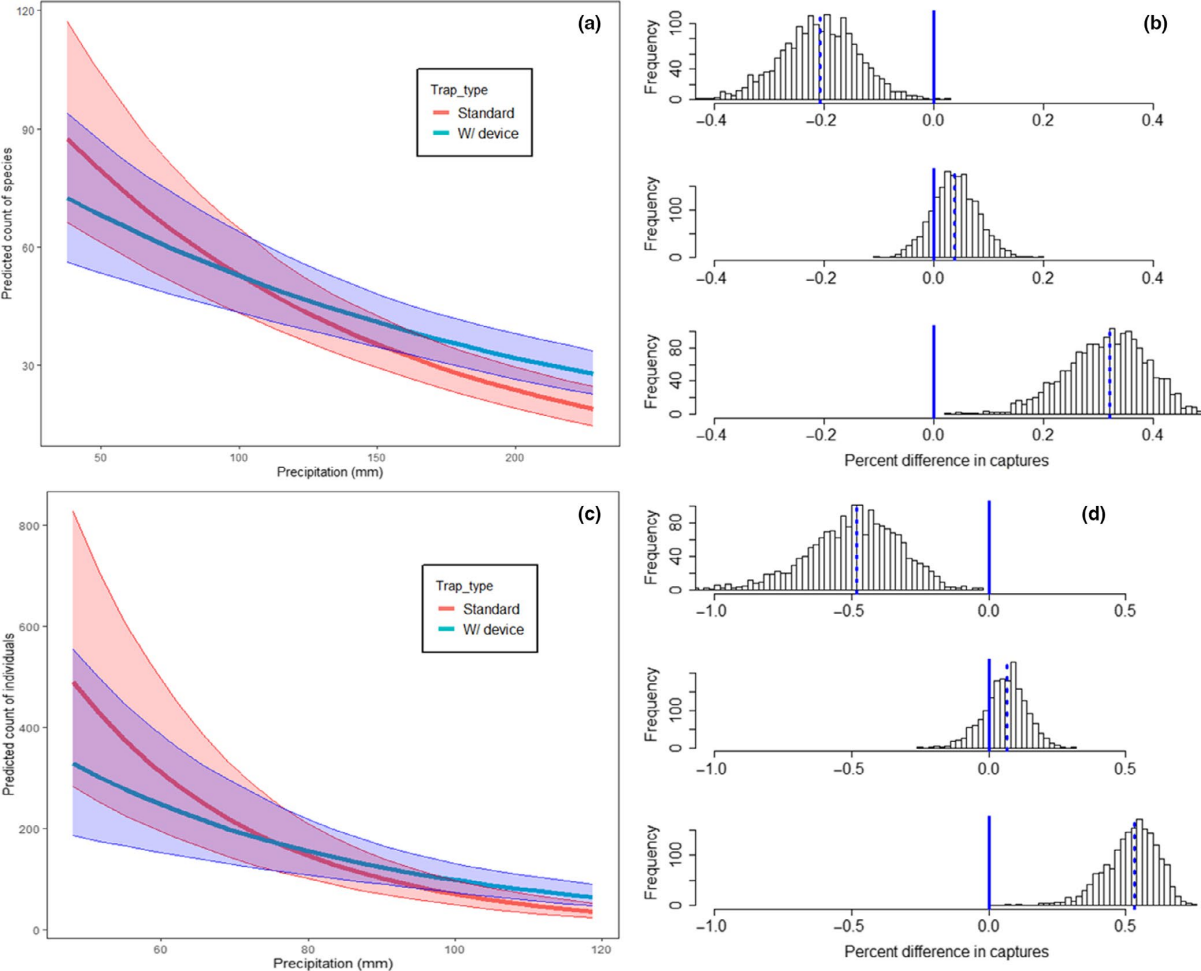
Predicted values for total number of beetle species (a) and individuals (c) captured by trap type across a precipitation gradient in Norway. Precipitation value represents total precipitation during a mean trap period of 38 days. Model predictions were made for mean values of effort during the first trap period of the season (late May to early July). Estimates of percent difference in number of captures using modified (w/device) versus standard traps (b,d) were estimated using random draws from the posterior model predictions (dashed line is the median value) for three levels of total precipitation across the trapping period: low (38 mm), medium (113 mm), and high (228 mm). Fewer insects are captured as precipitation increases, but standard traps catch more beetles than modified traps at low rainfall levels. At higher rainfall levels the modified traps perform better

Capture rates in both trap types responded negatively to precipitation, but this was most pronounced in the standard trap type (Figure 4a,c). When precipitation was low, standard traps caught more species (*μ* = 21.0%) and more individuals (*μ* = 49.3%) than did the modified traps (Figure 4b,d). At high precipitation levels, however, these numbers were reversed, with the modified traps catching more species (*μ* = 31.5%) and more individuals (*μ* = 51.9%) than standard traps. The same is true for natural forest‐indicator species (Fig. S2) and for large‐bodied species (Fig. S3). For small‐bodied species (Fig. S4), however, this pattern does not hold; rather, differences between traps are minimal but traps with the device appear to be slightly more effective.

## 4 DISCUSSION

Records from trap captures are important for answering many research questions, including estimating population densities, understanding interspecific interactions, and quantifying how species respond to habitat manipulation and climate change (Bowler et al., 2015; Burton et al., 2015; Ramsey et al., 2015). Capture records also represent nearly all that is known about many rare insect taxa. Here, we compared forest beetle capture rates between a standard flight intercept trap and a modified trap design that included a rainwater draining device and found that the relative effectiveness of these traps changed across a precipitation gradient, with the effect being stronger for larger bodied species.

Differences in detection among methods present a challenge that is generally recognized, and for which methods of correction are available (Guillera‐Arroita, 2017; Iknayan et al., 2014; MacKenzie et al., 2017; Roth et al., 2018), even if in practice such differences are perhaps too‐seldom quantified (Warton et al., 2015). However, we present evidence for a phenomenon that has received less attention though it is likely to be widespread—an interaction between climate and sampling method, such that relative effectiveness of trap types differs depending on precipitation levels. This interaction between trap type and climate means that a single correction factor, or factor variable in a model, can't adequately account for the differences in datasets collected using these two respective trap types. Furthermore, this bias differs with respect to species traits, affecting larger bodied species more strongly.

This challenge of climate‐dependent bias in capture records is troubling because precipitation levels vary on short time scales and are also exhibiting long‐term trends in many areas (Alexander, 2016). This is especially important to consider as long‐term monitoring programs for insects and other taxa are developed or upscaled (Åström et al., 2020; Montgomery et al., 2020). Climatic effects on capture rates mean that as precipitation and temperature patterns have changed and will change through time, capture records could be affected in ways that depend on both sampling methods and species traits. This could reduce or exaggerate apparent population trends through time, depending on any corresponding trends in the relevant climatic covariates.

Long‐term monitoring programs must combine consistent methods, detailed metadata regarding these methods (Montgomery et al., 2020), and experiments such as the one presented here to identify potential biases that can later be corrected for. One approach to doing this would be using an occupancy modeling framework (MacKenzie et al., 2017). If species traits that influence these detection relationships can be identified, as we have demonstrated here, and as is the case with other taxa (Garrard et al., 2013; Johnston et al., 2014), these traits can be used to better estimate detection for rare species. Methods should also be sought that are less climate‐dependent. This is difficult in forest insects, which are known to, for example, increase activity (and capturability) rates in warmer, dryer weather (Moser & Dell, 1979). Particular caution is advised when different sampling methods are not evenly distributed across space, time, and environmental gradients, such that any interactions between survey method and other covariates are confounded.

In the case of our standard and modified window traps, the likely mechanism for differing capture rates is intuitive—in dryer weather, beetles that enter the modified window trap have an extra barrier (the screen and funnel meant to divert rainwater) that they must crawl around in order to fall into the collection bottle. This means that some individuals might be able to fly back out of the trap and escape rather than falling into the collection bottle, and this may be more likely for larger beetles. In wetter weather, this loss of larger beetles no doubt still occurs, but losses in the standard traps may be even greater because collection vials sometimes overflow with rainwater in the standard traps, washing some individuals out of the trap. Reduced capture rates of larger beetles, and the resulting absence of information, is particularly concerning because large wood‐living beetles are rare and at increased risk for extinctions (Gillespie et al., 2017). Captures of natural forest‐indicator species differ in a similar way among the traps, although at low and moderate rainfall the magnitude of the effect is less, probably due to the smaller mean body size of indicator species in this study. The overall decline in capture rates in wet weather (for both trap types) is probably because beetle flight activity is reduced in wetter periods (Moser & Dell, 1979). A trap design is needed that excludes rainwater in a way that allows beetles to more easily fall into the collection bottle without escaping. The development of such a method is needed in order to overcome the challenges presented by an interaction between climate and sampling bias.

Detectability is but one of many issues that must be considered in robust sampling designs and meta‐analyses, and interactions between climate and sampling methods are unlikely to be the most important factor in all systems (Beissinger et al., 2016; Warton et al., 2015, 2016). In addition to the interaction between precipitation and climate, for example, our results suggest that changing the length or timing of sampling periods could have an even larger effect on captures rates. Nevertheless, we have shown that in some systems these captures biases could have effects that either mask or exaggerate trends as climate changes occur.

Our results not only provide evidence for changing sampling biases of a particular group of insect traps, but they also highlight the possibility of strong sampling biases that interact with climate patterns and species traits across a variety of sampling methods. This reinforces the fact that there will not always be a single “best” sampling method, even for a given taxonomic group and study objective. Choice of sampling methods may often involve tradeoffs, as in the case of our weather‐dependent effect of trap type. These concerns should be addressed explicitly in studies making use of capture data, and a detailed description of trap type and placement should be considered crucial metadata for all archived insect sampling datasets. When a single environmental factor with a large impact on detectability can be identified, as is the case with precipitation and flight intercept traps, we recommend choosing a combination of trap types that will allow for adequate sampling effort across the full range of climatic conditions that are likely for a given site. Careful study design and analyses will increase our ability to identify trends in insects (and other taxa) with higher confidence.

## 5 CONFLICT OF INTEREST STATEMENT

The authors have no conflicts of interest to declare.

## AUTHOR CONTRIBUTION

**Ryan C Burner:** Formal analysis (lead); Methodology (lead); Writing‐original draft (lead); Writing‐review & editing (lead). **Tone Birkemoe:** Conceptualization (equal); Funding acquisition (equal); Supervision (equal); Writing‐original draft (supporting); Writing‐review & editing (equal). **Siri Lie Olsen:** Conceptualization (supporting); Writing‐original draft (supporting); Writing‐review & editing (equal). **Anne Sverdrup‐Thygeson:** Conceptualization (equal); Funding acquisition (equal); Supervision (equal); Writing‐original draft (supporting); Writing‐review & editing (equal).

## Supporting information

Fig S1‐S4

## Data Availability

Data are available from the Dryad Digital Repository https://doi.org/10.5061/dryad.9w0vt4bd7
