# Supplementary material for: Sampling beetle communities: Trap design interacts with weather and species traits to bias capture rates
Source: Ecol Evol. 2020 Nov 18;10(24):14300–8. doi: 10.1002/ece3.7029 (PMC7771183; doi:10.1002/ece3.7029)
Supplement: Supplementary file 1 — Fig S1‐S4 [file ECE3-10-14300-s001.docx]

**SUPPLEMENTAL MATERIAL**


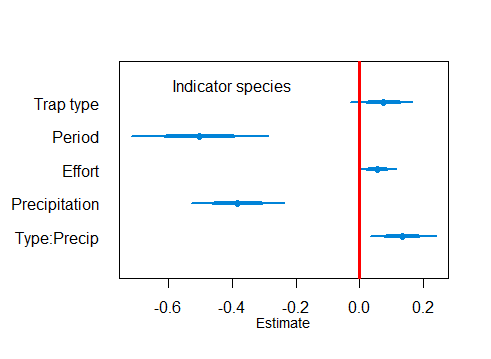


Figure S1. Coefficient estimates for the full model for indicator species capture rates from flight intercept traps placed in Norwegian boreal forests. Thick and thin blue lines show 95% and 68% posterior credible intervals, respectively. Five traps were placed in each of 20 sites, and intercepts for the sites were modeled as a random effect. Standard type traps were the intercept trap type, so the trap type parameter is an estimate for the traps that were modified to include a rainwater draining device.


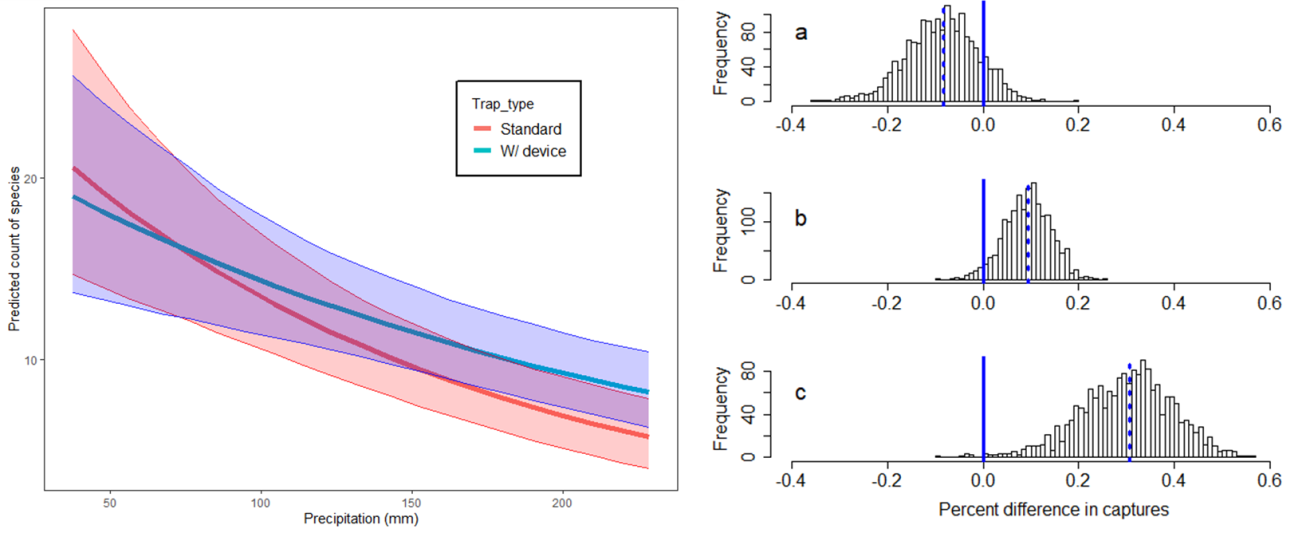


Figure S2. Predicted values for total number of natural-forest indicator beetle species (left) captured by trap type across a precipitation gradient in Norway. Precipitation value represent total precipitation during a mean trap period of 38 days. Model predictions were made for mean values of effort during the first trap period of the season (late May to early July). Estimates of percent difference in number of captures using modified (w/device) vs. standard traps (right) were estimated using random draws from the posterior model predictions (dashed line is the median value) for three levels of total precipitation across the trapping period: low (a; 38 mm), medium (b; 113 mm) and high (c; 228 mm). Fewer insects are captured as precipitation increases, but standard traps perform better than modified traps at low rainfall levels. At higher rainfall levels the modified traps perform better.


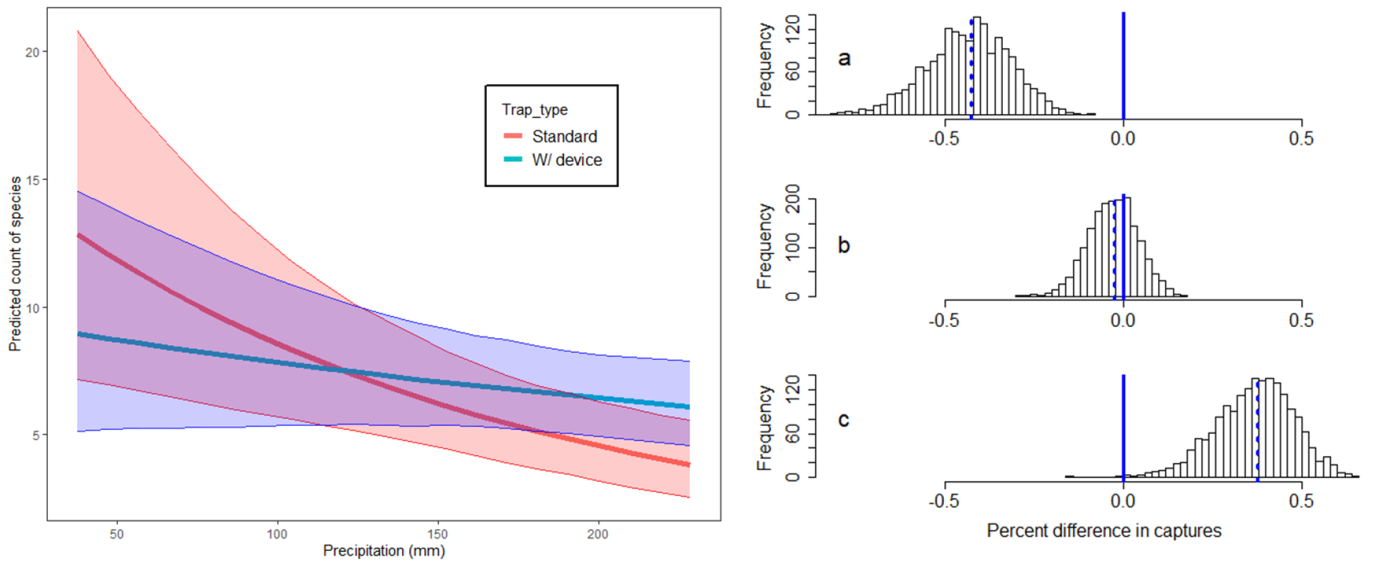


Figure S3. Predicted values for total number of large-bodied (length > 5.0 mm) beetle species (left) captured by trap type across a precipitation gradient in Norway. Precipitation value represent total precipitation during a mean trap period of 38 days. Model predictions were made for mean values of effort during the first trap period of the season (late May to early July). Estimates of percent difference in number of captures using modified (w/device) vs. standard traps (right) were estimated using random draws from the posterior model predictions (dashed line is the median value) for three levels of total precipitation across the trapping period: low (a; 38 mm), medium (b; 113 mm) and high (c; 228 mm). Fewer insects are captured as precipitation increases, but standard traps perform better than modified traps at low rainfall levels. At higher rainfall levels the modified traps perform better.


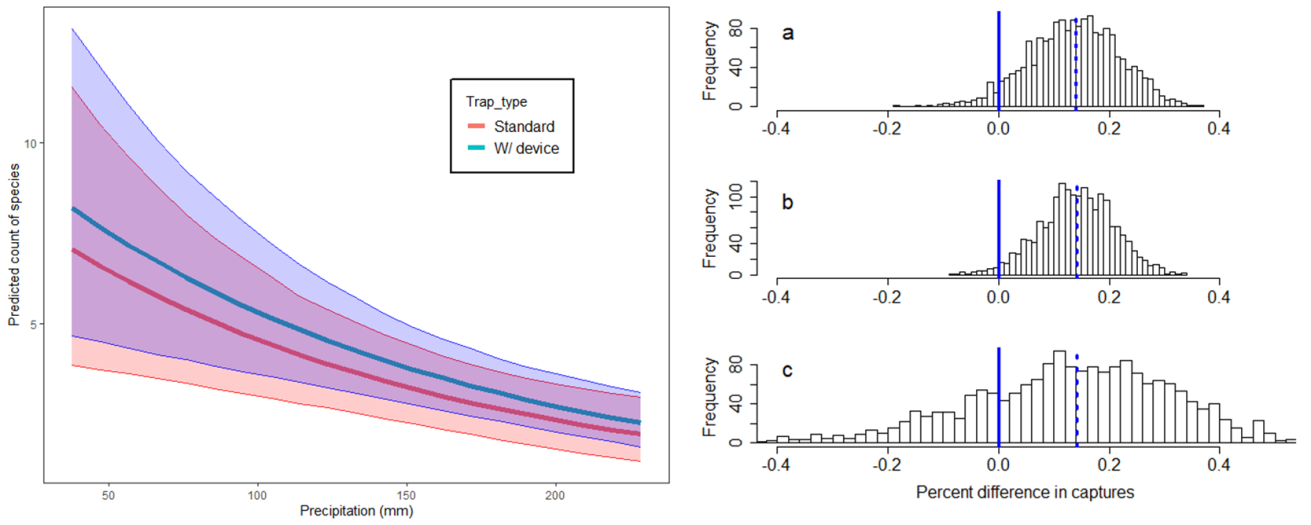


Figure S4. Predicted values for total number of small-bodied (length < 1.5 mm) beetle species (left) captured by trap type across a precipitation gradient in Norway. Precipitation value represent total precipitation during a mean trap period of 38 days. Model predictions were made for mean values of effort during the first trap period of the season (late May to early July). Estimates of percent difference in number of captures using modified (w/device) vs. standard traps (right) were estimated using random draws from the posterior model predictions (dashed line is the median value) for three levels of total precipitation across the trapping period: low (a; 38 mm), medium (b; 113 mm) and high (c; 228 mm). Fewer insects are captured as precipitation increases, and modified traps perform slightly better at most precipitation levels.
